# Locomotion Variations of Arch Index and Interlimb Symmetry in Shod and Barefoot Populations

**DOI:** 10.1155/2020/7610789

**Published:** 2020-05-28

**Authors:** Peimin Yu, Minjun Liang, Feng Ren

**Affiliations:** ^1^Faculty of Sports Science, Ningbo University, No. 818, Fenghua Road, Jiangbei District, Ningbo, Zhejiang, China 315211; ^2^Research Academy of Grand Health, Ningbo University, Ningbo, CN, China

## Abstract

The purpose of this study was to investigate the variations of arch index from static standing to dynamic walking and running; furthermore, the interlimb symmetry was checked in the two populations. A total of eighty male participants were recruited for this study, with forty habitually barefoot and forty habitually shod males, respectively. Arch index (AI) was calculated following the previously established “gold standard” measurement via contact areas recorded from EMED. Repeated measure analysis of variance (ANOVA) was employed to compare the difference between static and dynamic walking and running arch index. Paired-samples *t*-test and symmetry index (SI) were used to investigate the symmetry of the left foot arch index and right foot arch index. It was found that the dynamic arch index was significantly higher than the static arch index in barefoot and shod males, showing an increase from static weight-bearing standing to dynamic walking and running. However, interlimb (right-left) symmetry in the foot arch index was observed in the two populations. Dynamic changes of the arch index may provide implications that need to be considered while designing shoe lasts or insoles. Knowledge of the healthy arch index range reported from this study could also be used as a standard baseline to probe into foot and arch disorders.

## 1. Introduction

The human foot is a complex mechanical structure, consisting of 26 bones, 33 joints and relative muscles, tendons, and ligaments [1, 2]. Tarsal and metatarsal bones form the dome-like foot arches, including longitudinal and transverse arches, which enabled human upright bipedal locomotion [3]. Foot arch has been identified as a crucial structure contributing to the functions of the human foot and lower limb. Arch height could affect the foot pressure distribution during static standing and dynamic movements. A previous study has established the metrics to quantify the arch as per the plantar contact area, arch index (AI), which is calculated from midfoot area divided by the summation of forefoot area (excluding toes area), midfoot area, and rearfoot area [4]. The arch index has been widely used to identify the foot type according to the index value, as reported by Cavanagh and Rodgers [4], that the arch index less than 0.21 is classified as high arch foot, between 0.21 and 0.26 is classified as normal arch foot, and higher than 0.26 is indicated as low arch foot. What else, the arch index can be directly influenced by the foot contact areas, especially the midfoot. Wearing et.al [5] believed that body fat mass could affect the arch index, and increased fat mass could increase the contact area of the midfoot resulting in an increased arch index, which was reported as low arch or flat foot.

Foot types, including high arch, normal arch, and low arch, were previously reported with different structures and functional performance [6]. Whether the foot arch height influences the quality of life or not is still controversial [7, 8]. Low arch foot pronates excessively during running stance, while high arch foot is more rigid and inflexible. Insoles support based on foot plantar shape was believed to increase perceived comfort. Sun et.al [9] developed a three-quarter shoe insole in length, exhibiting altered pressure distribution over the plantar region, thus increasing comfort. Footwear companies designed functional shoes, such as arch support, for the cohort of different foot types, respectively. Traditionally, most arch support insoles were formed from the static weight plantar shape. However, assigning shoes as per foot types were proven not helpful to reduce the injury risk during the military training among soldiers [10–12]. Stearne et.al [13] provided direct evidence to support the energy-sparing spring theory of the arch and found that restricting arch compression increased the energy cost in the level run by hindering the arch's elastic energy storage, which was not effective during walking or incline running. Grau and Barisch-Fritz [14] found that compared to the static loading, most measurements such as length, width, and height increased during dynamic loading; nevertheless, all circumference measures decreased, which should be considered when designing the shoes or lasts.

Foot arch is an elastic structure, with arch height and index varying greatly during dynamic motions. It was reported that estimating dynamic medial longitudinal arch deformation using static foot metrics, such as foot posture index, medial longitudinal arch angle may not be reliable; thus, a new assessment was proposed [15]. Gait symmetry of able-bodied was widely hypothesized for the convenience of collecting and analyzing data. Whereas, angular changes of bilateral lower limb joints were asymmetric [16]. What is more, when analyzing stop-jump landing, some biomechanics parameters of dual-limb were not symmetric. Up to our knowledge, the symmetry about the arch index of the bilateral foot has not been investigated [17]. Thus, the objectives of this study were to (1) reveal the dynamic changes of the arch index during walking and running in shod and barefoot populations and (2) analyze the interlimb symmetry of the arch index between right and left feet. The hypothesis was that the arch index under dynamic conditions would increase significantly, and the arch index of both feet may be symmetric during walking and running.

## 2. Materials and Methods

### 2.1. Participant

The sample size was calculated before the test using G∗power version 3.0.10 (effect size = 0.5, *α* level = 0.05, power = 0.95). A total of 80 male participants joint the test, with forty habitually barefoot (age: 21.2 ± 1.6 yrs, height: 1.72 ± 0.07 m, mass: 66.2 ± 4.6 kg) and forty habitually shod (age: 24.5 ± 3.2 yrs, height: 1.74 ± 0.08 m, mass: 68.6 ± 7.5 kg) males. Barefoot participants, who ran and did physical activities in barefoot condition and wore slippers in their daily time since birth, were from South India (Kerala state). All these participants were students of Ningbo University and had significant differences in forefoot and toe morphology which had been highlighted in previous study [18]. Participants were physically active with habits of regular exercises. They were free of any lower extremity injuries in the past six months and presented no foot deformities prior to the test. Written informed consent was obtained from each participant, and they were informed of the requirements and procedures of this study, which was approved by the Ethical Committee from the research institute in the University (RAGH20170306).

### 2.2. Experimental Protocol

A Novel EMED pressure plate (Novel GmbH, Munich, Germany) was used to measure the static foot contact area while standing with feet shoulder-width apart (50% body weight distributed to both legs). Participants randomly selected to test right or left foot following the other. Before measuring the dynamic data using EMED, participants were required to warm up and practice for the familiarization with test environment and steps adjustment. The dynamic walking and running foot contact areas were collected on a 20-meter runway, with the Novel EMED pressure plate fixed in the middle. To ensure normal gait performance, participants walked and ran at a self-selected speed. Four successful trials of each left and right foot landing were recorded during walking and running. All the tests were carried out under barefoot condition and well-designed based on previous guidelines [19].

### 2.3. Data Processing

The contact area between the foot plantar surface and the EMED pressure plate was processed and obtained from Novel Database software. The plantar region was classified into rearfoot, midfoot, forefoot, and toe areas, with contact areas of rearfoot, midfoot, and forefoot used in this study. Arch index for the right and left limb was calculated using Equation (1) as per previously published protocol [4, 20].

(1) $$\begin{array}{c} \text{Arch Index}=\frac{\text{Midfoot contact area}}{\left(\text{Forefoot}+\text{Midfoot}+\text{Rearfoot}\right)\text{ contact area }}, \end{array}$$

To test the interlimb symmetry of the arch index, an established symmetry index (SI) Equation (2) [21] was used to calculate the right-left symmetry (AI*R* arch index of right foot;AI*L* arch index of left foot)

(2) $$\begin{array}{c} \text{Symmetry Index}=\frac{\;|\;{\text{AI}}_{R}-{\text{AI}}_{L}\;|\;}{0.5\left({\text{AI}}_{L}+{\text{AI}}_{R}\right)}\times 100\%, \end{array}$$

### 2.4. Statistical Analysis

Average of four trials were taken before applying the inferential statistics, which would ensure a reduction in intertrial errors in the performance outputs reported. SPSS Statistics version 25.0 (SPSS, Chicago, IL, US) was used for the statistical analysis. Descriptive statistics (mean and standard deviation) for the contact areas of all the trials were calculated. To analyze the significance between the static standing and dynamic (walking and running) arch index, repeated measure analysis of variance (ANOVA) was employed. Paired-sample *t*-test and symmetry index were used to investigate the difference and symmetry of left and right foot arch index. A two-sided confidence interval with an alpha level of 0.05 was used to define significance.

## 3. Results

### 3.1. Arch Index during Standing, Walking, Running from EMED in Shod and Barefoot Males

**Figure 1:**
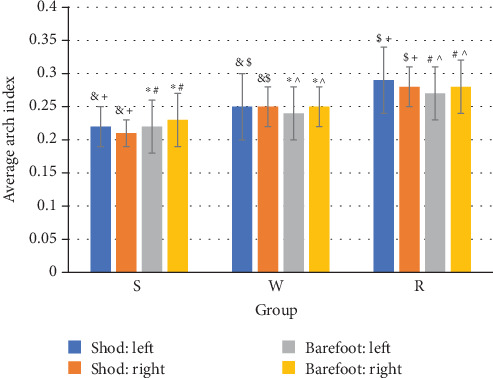
Mean (±SD) arch index from EMED while standing (S), walking (W), and running (R) in barefoot and shod males.

From static standing to dynamic walking and running, the average arch index showed significant increasing trend (Figure 1). With respect to the left foot arch index, a 1.8% increase was observed in walking when compared to standing (*p* = 0.007, Table 1). While in running, a significant increase of 3% was found in contrast to walking (*p* = 0.001, Table 1). The same change was found in the arch index of the right foot during walking, which produced a significant increase of 2% (*p* = 0.007, Table 1) compared to standing. While comparing running with walking, an increase of 3% (*p* = 0.002, Table 1) was noted.

### 3.2. Arch Index during Standing, Walking, Running from EMED in Shod Males

Similar to the changes in barefoot populations, significant increases of the arch index were observed from standing to walking and running in shod males (Figure 1). When comparing the running task with walking task, the arch index value significantly increased by 4% and 3% for left and right feet (*p* = 0.007, *p* = 0.001, Table 1), respectively.

### 3.3. Symmetry of Arch Index between Left and Right Feet in Barefoot Males

The results of the paired-sample *t*-test for left and right feet under different conditions showed no significant difference (Table 2). Further, using the symmetry index to demonstrate the symmetry of left and right feet, which has been proved to be the most sensitive method to assess interlimb symmetry among healthy individuals ^15^. When the value of SI = 0, it indicates complete symmetry; when the value of SI ≥ 100%, it indicates asymmetry [21]. The result showed the left feet and right feet were symmetric (Table 2).

### 3.4. Symmetry of Arch Index between Left and Right Feet in Shod Males

The results of the paired-sample *t*-test for left and right feet under different conditions in shod males were similar to those in barefoot, which showed no significant difference. Different from barefoot males, the left foot arch index was slightly greater than right foot arch index, but this difference was not significant. Further, the symmetry of the left and right foot arch index was proven owning to the symmetry index value was 0.02% and 0.03% during walking and running, respectively.

### 3.5. Comparison of the Arch Index in Barefoot and Shod during Walking and Running

The result of the independent-sample *t*-test showed no difference of arch index between barefoot and shod males during walking and running (Table 3).

## 4. Discussion

The main finding of this study was that the arch index increased greatly from static weight-bearing standing to dynamic walking and running conditions but showed good symmetry between left and right feet during walking and running tasks, which are supported by our hypothesis.

Arch index based on footprint has been proven to as an effective method to reflect the arch height [20]. However, the body composition could be a confounding factor while calculating the arch index based on footprint. Wearing et.al [5] reported that fat mass was associated with the midfoot contact area. The greater fat mass would increase the midfoot contact area, which resulted in a higher arch index (flat foot). On the contrary, fat-free mass affected the total foot contact area, especially the forefoot and hindfoot area; moreover, the fat-free mass was not related to the midfoot contact area. Another study also found a medial longitudinal arch of obese individuals collapsed due to greater downward vertical forces [22]. Genevieve et.al [23] illustrated that ageing was significantly associated with changes in foot characteristics and resulted in redistributed planter loading patterns. The normal range of the arch index in older people is slightly different to the young cohort. The normal arch index is identified between 0.21 and 0.28, exceeding or less than this range is considered to be the low or high foot arch [24]. Bertsch et.al [25] found that the shape and loading characteristics of a child's foot changed significantly as long as the child started to walk. Furthermore, Onodera et.al [26] demonstrated that between the age of 3 and 4 years, the incidence of the low arch was high, and between the age of 4 and 5 years, the longitudinal arch acquired an adult-like shape gradually. Tong and Kong [27] suggested not to measure static footprint on children due to the light body weight which may result in incomplete footprint contact.

Different from the above studies and findings, this study included young and physically active individuals with different shoe-wearing habits (shod or barefoot), all in the same age and weight group, thus alleviating the influence from age, weight, and potential pathological conditions.

While referring to the arch index, the static arch index was mostly discussed in the past studies. However, foot parameters change under the dynamic conditions. Mathieson et.al [28] found footprint parameters calculated from dynamic footprints were generally higher than those from static footprints. Cavanagh and Rodgers [4] demonstrated the arch index values increased from walking to running by about 10%. Grau and Barisch-Fritz [14] that most length, width, height, and angle measurements increased significantly, while all the circumference measures decreased during dynamic tasks. All these changes should be taken into the consideration when designing and manufacturing the shoe lasts or insoles. Thus, assigning shoes based on static plantar foot shape to increase comfort and prevent injury may not be perfectly effective. Previous studies have proven that assigning shoes based on static foot shape has little influence on injury reduction in military basic training [10, 12]. Another merit of the present study was revealing changes of foot plantar shape during static (standing) and dynamic (walking and running) conditions via illustrating the arch index. The results of this study showed high consistency with previous findings. Our results have demonstrated dynamic arch index would be significantly greater than the static arch index. What else, under dynamic conditions, the running arch index increased significantly comparing to the walking arch index. Foot arch, as a support structure, plays an important role during the movements. Arch compression/recoil, functioning like a spring, could reduce the energy cost [13]. While walking, the load to the longitudinal arch was greater than standing, so did the extent of compression for arch, and consequently midfoot contact area increased, which finally lead to increased arch index value. Similarly, during running, the load on the arch was greater than walking, and the degree of arch compression was greater, so the arch index was significantly higher than walking. As mentioned above, the dynamic arch index was significantly different from the static arch index, which could partly explain the reason why assigning shoes based on static foot plantar shape had little influence on reducing injury risks. Dynamic arch index changes could be a factor that may need consideration while designing and manufacturing shoe last and insoles.

Comparing differences between static and dynamic tasks, the arch index of the right foot was found to be slightly greater than that of the left foot. Using the symmetry index and paired-sample *t*-test to check whether this distinction was significant, it was demonstrated the difference between right and left foot arch index did not have statistical significance. The symmetry index scores showed similar results that arch index of the left foot was symmetry to that of the right foot. The reason why the right foot arch index slightly higher than the left foot arch index may be the right leg is the dominant leg for most populations. Whether under static or dynamic conditions, individuals tend to bear more weight on the dominant leg. Additionally, the right leg provided more energy for movements, thus loading compression to the longitudinal arch of the right foot was slightly greater than that of the left foot. However, the arch index of the left foot and the right foot is symmetry in our results. This finding may be useful for customized shoe or insole development for high-demand individuals or patients.

Limitations in this study should be considered and included into further investigation. The current study only recruited healthy individuals without foot deformities or malfunctions, which was aimed to compare this functional difference in barefoot and shod populations with different shoe-wearing habits. However, findings from this study could be used as baseline data to investigate pathological feet with arch malfunctions.

## 5. Conclusions

This study found that the dynamic arch index was significantly higher than the static arch index in barefoot and shod males, showing an increase from static weight-bearing standing to dynamic walking and running. These changes may provide implications that need to be considered while designing shoe lasts or insoles. Knowledge from this study could also be used as a standard baseline to probe into foot and arch disorders.

## Figures and Tables

**Table 1:** Comparisons of arch index in left and right feet from standing to walking and running in shod and barefoot males.

Population			Standing	Walking	Running
Barefoot	LFAI (cm^2^)	Mean	0.22∗^#^	0.24∗^^^	0.27^#^^
		SD	0.04	0.04	0.04
	RFAI (cm^2^)	Mean	0.23∗^#^	0.25∗^^^	0.28^#^^
		SD	0.04	0.03	0.04

Shod	LFAI (cm^2^)	Mean	0.22^&+^	0.25^$&^	0.29^$+^
		SD	0.03	0.05	0.05
	RFAI (cm^2^)	Mean	0.21^&+^	0.25^$&^	0.28^$+^
		SD	0.02	0.03	0.03

Notes: LFAI: left foot arch index; RFAI: right foot arch index; SD: standard deviation; barefoot population: ∗significant difference between standing and walking; ^#^significant difference between standing and running; ^^^significant difference between walking and running. Shod population: ^&^significant difference between standing and walking; ^+^significant difference between standing and walking; ^**$**^significant difference between walking and running (*p* < 0.05).

**Table 2:** Comparisons of arch index in left and right feet from standing to walking and running in shod and barefoot males.

Population		Mean	SD	95% CI	Sig.	SI
Barefoot	Pair 1 sl-sr	-0.00	0.04	-0.02-0.01	0.55	0.02%
	Pair 2 wl-wr	-0.01	0.03	-0.02-0.00	0.09	0.04%
	Pair 3 rl -rr	-0.01	0.03	-0.02-0.00	0.24	0.02%

Shod	Pair 1 sl-sr	0.00	0.03	-0.00-0.03	0.36	0.02%
	Pair 2 wl-wr	0.00	0.04	-0.01-0.02	0.57	0.02%
	Pair 3 rl -rr	0.01	0.04	-0.00-0.02	0.17	0.03%

Notes: SI: symmetry index; sl/r: left/right foot while standing; wl/r: left/right foot while walking; rl/r: left/right foot while running.

**Table 3:** Comparison of the arch index in barefoot and shod during walking and running.

	Condition	95% CI	MD	SED	Sig.
LFAI	Walking	-0.01-0.03	0.01	0.01	0.029
	Running	-0.00-0.04	0.02	0.01	0.079

RFAI	Walking	-0.02-0.01	0.01	0.01	0.811
	Running	-0.01-0.02	0.01	0.01	0.647

Notes: MD: mean difference; SED: Std. error difference.

## Data Availability

The data used to support the findings of this study are available from the corresponding author upon request.
